# Dictionary of Medical Terms (English—French)

**Published:** 1899-12

**Authors:** 


### Reviews of Books.

---

**Dictionary of Medical Terms (English—French).** By H. DE MÉRIC. Pp. vi., 394. London: Baillière, Tindall and Cox. 1899.

The average Englishman's knowledge of the French language, consisting for the most part of an imperfect acquaintance with a few classical authors, is of little value to him in the practice of medicine. We remember well when a few years ago attending a child with croup how great we found the difficulty in explaining to the French parents the use of a steam-kettle. M. de Méric has come to the rescue of those who suffer embarrassment in speaking or reading medical French. This English-French dictionary includes many terms derived from the cognate sciences as well as those belonging to medicine proper, and is especially rich in the matter of surgical terms. An exceedingly useful book has been added to our lexicographical store.

